# Comment on the publication of Amiryousefi et al. (2017)

**DOI:** 10.1080/23802359.2017.1413316

**Published:** 2017-12-12

**Authors:** Eszter Virág, Erzsébet Nagy, János Taller

**Affiliations:** Department of Plant Science and Biotechnology, University of Pannonia, Georgikon Faculty, Keszthely, Hungary

**Keywords:** *Ambrosia artemisiifolia*, chloroplast genome

## Abstract

This announcement describes corrections and comments to the paper entitled ‘The plastid genome sequence of the invasive plant common Ragweed (*Ambrosia artemisiifolia*, Asteraceae)’ by A Amiryousefi, J Hyvönen, P Poczai.

Recently, a paper by Amiryousefi et al. (2017) appeared in this journal claiming publication of the common ragweed (*Ambrosia artemisiifolia*) plastid genome sequence. The paper contains no supporting new data of the authors’ claims. Detailed data on the chloroplast genome structure was published by Nagy et al. (2017).

The complete chloroplast genome sequence of *A. artemisiifolia* is available in NCBI GenBank under accession number MF362689; https://www.ncbi.nlm.nih.gov/nuccore/MF362689 deposited by Virág et al. (2016). Based on the GenBank data the structure of *A. artemisiifolia* complete chloroplast genome is the following:

**Table:** 

*A. artemisiifolia cp genome*	MF362689
Complete genome size (bp)	152,215
Number of tRNA	30
Number of rRNA	4
Number of protein coding genes	80

Further, the publication of Virág et al. (2016) that refers to the deposition of raw reads and transcriptome datasets of common ragweed reproductive organs in NCBI TSA and SRA databases was cited in a misinterpreted context.
